# Stroke as a rare complication of scorpion stings: A systematic review and analysis

**DOI:** 10.1016/j.toxcx.2024.100205

**Published:** 2024-08-30

**Authors:** Jorge Vasconez-Gonzalez, Karen Delgado-Moreira, Esteban Gamez-Rivera, María Belen Lopez-Molina, Fredy Lizarazo Davila, Juan S. Izquierdo-Condoy, Esteban Ortiz-Prado

**Affiliations:** a One Health Research Group, Faculty of Health Science, Universidad de Las Americas, Quito, Ecuador; b Interinstitutional Internal Medicine Group (GIMI 1), Universidad Libre, Cali, Colombia

**Keywords:** Scorpion sting, Scorpionism, Ischemic stroke, Hemorrhagic stroke

## Abstract

Approximately 1 million scorpion stings are recorded annually worldwide, resulting in 3000 deaths. Scorpion venom has various effects on the human body, with neurological complications occurring in about 2% of cases. Among these complications, stroke—whether ischemic or hemorrhagic—is particularly significant. A systematic literature review was conducted through a bibliographic search using key terms in the PubMed, Scopus, Scielo, Latin American and Caribbean Literature in Health Sciences (LILACS) and Google Schoolar databases without date restrictions. Articles related to stroke due to scorpion stings in Spanish, English, and Portuguese were included. Our protocol was registered in PROSPERO. A total of 24 articles met the inclusion criteria for this review. The primary neurological symptoms caused by scorpion stings include hemiplegia, hemiparesis, seizures, and limb weakness. Stroke should be suspected in the presence of these symptoms, as scorpion stings can lead to both hemorrhagic and ischemic strokes in both adults and pediatric populations. While stroke is a rare complication of scorpion stings, it is crucial to consider this diagnosis in patients presenting with neurological symptoms, necessitating the use of computed tomography or magnetic resonance imaging if stroke is suspected.

## Highlights

• Over 1 million people are stung by scorpions yearly, causing significant injuries.
• Scorpion venom disrupts the autonomic nervous system, causing severe symptoms.
• Scorpion stings can cause 2% central nervous system and 8% cerebrovascular issues.
• Neurotoxins in venom cause hypertension, tachycardia, and myocardial dysfunction.
• Early antivenom and prazosin administration reduce complications and mortality.

## Graphical abstract

**Figure:**
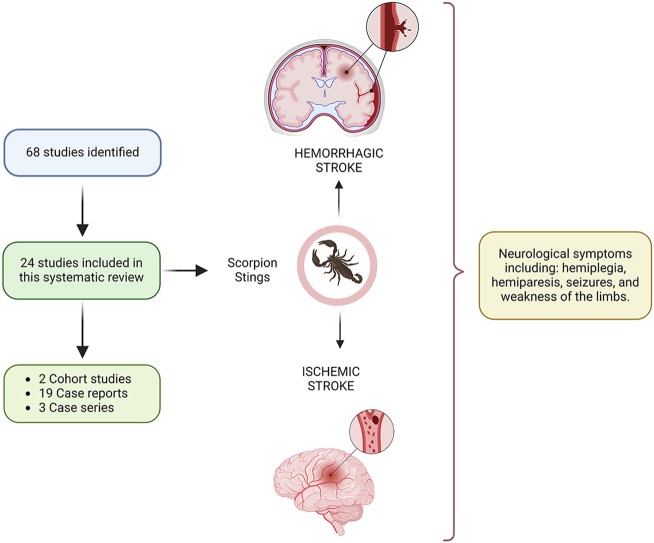

## 1 Introduction

There are at least 2500 species of scorpions on the planet, and they are part of most ecosystems worldwide (Lira et al., 2023). Scorpions are venomous arthropods, members of the class Arachnida in the order Scorpiones (Cloudsley-Thompson, 1993). Based on geographic distribution, at least 50 species are clinically significant for humans (Naranjo et al., 2021a; Santos et al., 2016). As humans inhabit these regions, coexistence with scorpions is common, leading to frequent interactions and an increased risk of sting-related injuries and fatalities (Petricevich, 2010). Annually, scorpion stings affect over 1 million people, resulting in numerous injuries and hospitalizations, with at least 3000 cases resulting in death worldwide (Gopalakrishnakone et al., 2015; Naranjo et al., 2021a).

Scorpion venom is a complex cocktail of toxins, with neurotoxins being particularly prominent. Other components include cardiotoxins, nephrotoxins, hemolytic toxins, phosphodiesterases, phospholipases, glycosaminoglycans, histamine, serotonin, tryptophan, cytokine releasers, bradykinin-enhancing peptides, toxins with healing properties, and enzymes such as hyaluronidase (Ochoa-Andrade et al., 2022). Depending on the species, scorpions can produce neurotoxic, cardiotoxic, and hemolytic toxins, causing effects ranging from mild to moderate, including numbness, neuralgic or stabbing pain, general discomfort, dizziness, irritability, and migraines (Ochoa-Andrade et al., 2022), However, some toxins are potent enough to cause severe reactions in humans, such as breathing difficulties, severe inflammatory responses, seizures, loss of consciousness, coma, and even death (Fernández-Bouzas et al., 2000).

Due to their widespread distribution, scorpion stings represent an often forgotten public health issue (Baleela et al., 2024). These species are found in tropical and subtropical regions, including Central and South America, North Africa, the Middle East, and India (Nejati et al., 2018). Notably, in Morocco, scorpion stings are the leading cause of high morbidity and mortality, representing the most common form of poisoning (Imad et al., 2023). Similarly, in India, scorpion stings have a reported mortality rate of 1.5% (Gadwalkar et al., 2006). In South America, approximately 16.36 cases per 100,000 inhabitants are reported annually, with a mortality rate of 0.05 per 100,000 inhabitants. In Mexico, the incidence is 233.64 per 100,000 inhabitants, while in the Amazon River Basin, there are 22.15 cases per 100,000 inhabitants per year, with a mortality rate of 0.03% (Ochoa-Andrade et al., 2022; Trinidad-Porfirio et al., 2023).

Among severe cases, scorpion toxins can not only affect the nervous system but also target specific organs such as the heart and brain (Godoy et al., 2021.). In rare instances, ischemic or hemorrhagic strokes have been reported as consequences of scorpion stings, leading to neurological complications in 2–5% of cases (Naranjo et al., 2021a). Although infrequent, stroke due to scorpion envenomation is mentioned in various prevention guides and first aid manuals (Kalkonde et al., 2018).

The public health significance of scorpion stings and their consequences has been well-documented (Lacerda et al., 2022). However, the previously anecdotal link between scorpion stings and stroke is now recognized as a more relevant global health issue due to the potential for ischemic or hemorrhagic complications in the brain. These can lead to severe neurological sequelae and even death (Naranjo et al., 2021b).

To address this, we have comprehensively collected data on strokes caused by scorpion stings. Our goal is to provide global readers and the medical community with a detailed guide to understanding these rare but serious cases. This review aims to enhance our understanding and management of this severe consequence of scorpion envenomation, ensuring better preparedness and response in medical practices worldwide.

## 2 Methodology

### 2.1 Research question

Can scorpion stings cause ischemic or hemorrhagic strokes?

### 2.2 Study design

We conducted a systematic review that included cross-sectional studies, case-control studies, descriptive observational studies, case reports, and case series. Excluded from this review were systematic reviews, meta-analyses, narrative reviews, letters to the editor, editorials, and opinion articles. The methodology followed the PRISMA (Preferred Reporting Items for Systematic Reviews and Meta-Analyses) guidelines, which are recommended for conducting systematic reviews and meta-analyses. Our review protocol is registered in PROSPERO under the registration ID: CRD42024555500.

### 2.3 Search strategies

Bibliographic searches were conducted in Spanish, English, and Portuguese to encompass the widest range of available information. We reviewed the PubMed, Scopus, Scielo, Latin American and Caribbean Literature in Health Sciences (LILACS) and Google Schoolar databases without time restrictions to cover extensive literature. Additionally, reference lists of identified articles were reviewed to access potentially relevant studies.

The following search strategy with key terms was used for the bibliographic search: in English: ((“scorpion sting” OR “scorpion envenomation” OR “scorpionism”) AND (“stroke” OR “cerebrovascular disease” OR “ischemic stroke” OR “hemorrhagic stroke")); in Spanish: ((“picadura de escorpión" OR “escorpionismo” OR “intoxicación por escorpión") AND (“accidente cerebrovascular” OR “enfermedad cerebrovascular” OR “accidente cerebrovascular isquémico" OR “accidente cerebrovascular hemorrágico")); and in Portuguese: ((“picada de escorpião" OR “envenenamento por escorpião" OR “escorpionismo”) AND (“acidente cerebrovascular” OR “doença cerebrovascular” OR “acidente vascular cerebral isquémico" OR “acidente vascular cerebral hemorrágico")).

### 2.4 Studies selection

#### 2.4.1 Inclusion criteria

- All manuscripts involving human subjects.
- Manuscripts with “scorpion sting” or “scorpion envenomation” or “scorpionism” in the title or abstract and including “stroke” or “cerebrovascular disease” or “ischemic stroke” in the title or abstract.

#### 2.4.2 Exclusion criterion

- Animal studies.
- Studies evaluating bites or stings of other arachnids or insects.
- Studies examining neurological complications other than stroke.
- Studies analyzing cardiovascular complications other than stroke.

**Fig.1:**
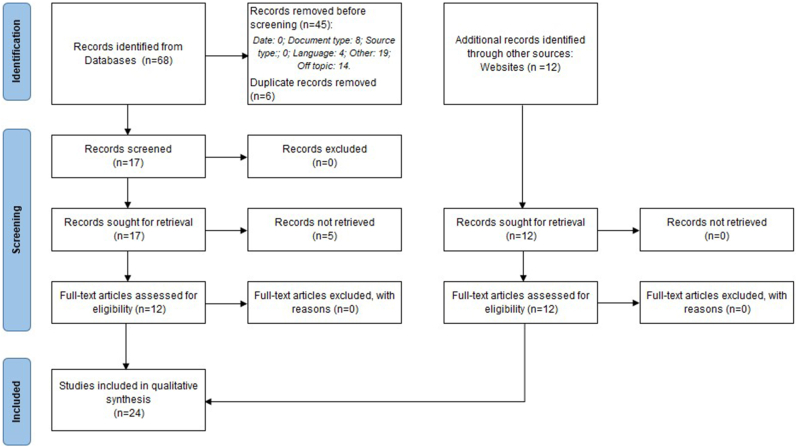
PRISMA Flow chart of selection of studies.

The initial bibliographic search yielded 68 papers. In the first screening phase, 51 studies were excluded. Of the remaining 17 papers, 5 were excluded due to retrieval issues. Finally, 12 papers underwent a full review and were included. Additionally, 12 manuscripts from other sources (websites) were included after a full review, totaling 24 studies in this investigation. Fig. 1 illustrates the selection process based on the PRISMA flow chart for the studies analyzed in this manuscript.

### 2.5 Bias assessment

To minimize bias, data extraction was independently performed by JEV, KD, and EGR at different times. Discrepancies in data collection from primary studies were resolved through discussion and consensus.

### 2.6 Data synthesis

We conducted a comprehensive review of all manuscripts meeting the inclusion criteria. Quantitative analysis was performed using the Newcastle-Ottawa Quality Assessment Scale for cohort and case-control studies, the Joana Briggs Institute (JBI) critical appraisal checklist for analytical cross-sectional studies, and the JBI critical appraisal checklist for case reports and case series. The studies evaluated with these scales were of moderate to high quality. Information from the manuscripts was then organized and synthesized into tables for better clarity and analysis.

This process ensured a thorough evaluation of the quality and relevance of the included studies, providing a robust foundation for our conclusions and recommendations.

## 3 Results

**Fig.2:**
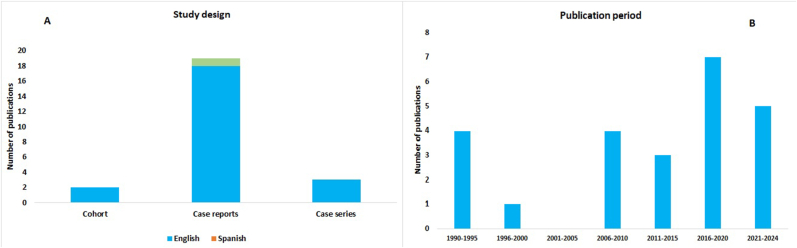
Distribution of the publications of stroke due scorpion sting**. A.** Publication types, in English or in Spanish; **B.** Period of publication of the papers, divided into periods of 5 years.

A total of 24 articles met the inclusion criteria for this systematic review. Table 1 presents the main characteristics of the included studies. Quality assessment revealed that 2 cohort studies were of good quality (see Table S1), while 19 case reports were included, with 16 rated as high quality and 3 as moderate quality (see Fig. 2A and Table S2). Additionally, 3 case series were included, with 2 classified as high quality and 1 as moderate quality (see Table S3). All identified records were published between 1991 and 2024 (Fig. 2B).

**Table 1:** Main Characteristics of Included Studies: This table presents a detailed overview of the 24 studies included in the systematic review, highlighting key characteristics such as author, year, study design, population, scorpion type, and major findings related to scorpion stings and their clinical impact.

Author	Year	Study design	Population	Scorpion type	Result
Annobil et al. (Annobil et al., 1991)	1991	Case report	1	*Nebo hierichonticus*	Cranial CT (8 days after admission) showed bilaterally symmetrical multiple hyperdense areas with intense enhancement in the cerebellar and cerebral hemispheres consistent with multiple hemorrhages.
Groswasser et al. (Groswasser et al., 1991)	1991	Case report	1	*Leiurus quinquestriatus*	A CT scan disclosed mainly biopercular infarcts.
Nagaraja et al. (Nagaraja et al., 1994)	1994	Cohort	43	N/A	2 patients present scorpion sting as the cause of stroke.
Sousa et al. (Sousa et al., 1995)	1995	Case report	1	*Tityus Sp.*	CT showed presence of a subcortical encephaloma-lacia lesion in the fronto-temporoparietal region of the right cerebral hemisphere that does not have a mass effect.
Fernández-Bouzas et al. (Fernández-Bouzas et al., 2000)	2000	Case report	2	N/A	Brain infarct secondary to a scorpion sting in children who lived in small, remote towns with poor communications.
Udayakumar et al. (Udayakumar et al., 2006)	2006	Case series	50	N/A	Cerebrovascular involvement was noted in four patients (8%). Hemorrhagic stroke was noted in two patients (4%) and thrombotic stroke was noted in two patients (4%).
Jain et al. (Jain et al., 2006)	2006	Case report	1	N/A	Scorpion sting followed by multiple cerebral and cerebellar watershed infarctions.
Bhattacharya et la. (Bhattacharya et al., 2008)	2008	Case series	42	N/A	Hemorrhagic stroke was noted in two patients (4.8%) and thrombotic stroke was noted in one patient (2.4%).
Bouaziz et al. (Bouaziz et al., 2008)	2008	Cohort	951	N/A	Brain CT (n = 10) was abnormal in 90% of cases. The more usually observed lesions were brain ischemia at 5 patients (50%).
Sığırcı et al. (Sığırcı et al., 2014)	2014	Case report	1	*Leiurus quinquestriatus*	Cerebellar and cerebral infarctions with corpus callosum involvement and bilateral cerebral atrophy with subdural hemorrhage.
Prasad et al. (Prasad et al., 2014)	2014	Case report	1	N/A	Cranial CT showed infarction in right and left frontoparietal lobes.
Eze et al. (Eze et al., 2014)	2014	Case report	1	N/A	Elderly woman with hemorrhagic stroke as a complication of a sting from a scorpion which was killed and thereafter ingested.
Bucaretchi et al. (Bucaretchi et al., 2016)	2016	Case report	1	*Tityus serrulatus*	Fatal envenomation involving multiple, extensive brain infarcts in a patient with a previous diagnosis of ET who was stung by *Tityus serrulatus*.
Nataraja et al. (Nataraja et al., 2016)	2016	Case report	2	*Hottentotta tamulus*	Case 1: MRA with DWI of brain showed an acute infarct in the right capsuloganglionic region. Case 2: Non-contrast computed tomography of brain showed acute hemorrhage in left frontal region.
Reddy et al. (Reddy et al., 2017)	2017	Case report	1	*Hottentotta tamulus*	MRI of brain revealed massive left MCA territory infarct.
Mishra et al. (Mishra et al., 2018)	2018	Case series	5	N/A	Patients had evidence of CVI in imaging.
Nagar et al. (Nagar et al., 2018)	2018	Case report	1	N/A	Non-contrast CT head showed multiple infarcts involving bilateral cerebellar hemisphere, bilateral occipital lobes, medulla and Pons on right side, right temporal lobe, and right thalamus, left parietal lobe.
Bordon et al. (Bordón et al., 2018)	2018	Case report	1	*Tityus trivitatus*	Neurological deterioration is detected secondary to intracerebral hemorrhage.
Majumdar et al. (Majumdar et al., 2020)	2020	Case report	1	*Mesobuthus tamulus*	Non-contrast CT revealed intracerebral hemorrhages in left temporoparietal lobe extending into left basal ganglia with intraventricular extension.
Naranjo et al. (Naranjo et al., 2021a)	2021	Case report	1	Scorpion of genus *Tityus*	A scorpion sting in a child who received antivenom immunotherapy 2 days after the sting and who subsequently experienced an ischemic brain stroke.
Ravi & Kanda (Ravi and Kandan, 2023)	2023	Case report	1	N/A	Schemic stroke in the occipital region secondary to scorpion causing blindness.
Imad et al. (Imad et al., 2023)	2023	Case report	1	N/A	Ischemic stroke in the territory of the left middle cerebral artery, with individualization of a patch of cortico-subcortical hypodensity, systematized fronto-parieto-temporal on the left side.
Uysal et al. (Uysal et al., 2023)	2023	Case report	1	N/A	Noncontrast CT showed the hyperdense foci in both posterior parietal lobes, suggestive of SAH.
Aslanyavrusu et al. (Aslanyavrusu et al., 2024)	2024	Case report	1	N/A	Transient ischemic attack after scorpion sting.

CT: computed tomography, SAH: subarachnoid hemorrhage, MCA: middle cerebral artery, ET: essential thrombocythemia, MRA: Magnetic resonance angiogram, DWI: diffusion weighted imaging, CVI: cerebrovascular injury.

To provide a comprehensive overview of the relationship between scorpion stings and cerebrovascular incidents, we reviewed 24 studies that met our inclusion criteria. These studies encompass various designs, including case reports, cohort studies, and case series, illustrating the diverse clinical presentations and outcomes associated with scorpion envenomation. The following table summarizes the key characteristics and findings of each study, highlighting the types of scorpions involved, the study populations, and the specific cerebrovascular effects observed. This compilation aims to underscore the significant yet often overlooked impact of scorpion stings on human health, particularly regarding their potential to induce ischemic and hemorrhagic strokes (Table 1).

### 3.1 Overall description and characteristics of included studies

We identified cases from 9 countries across different continents (Asia, Africa, and South America). The majority of articles originated from India (n = 11), followed by Turkey (n = 3). Most cases occurred in males, with a total of 14 articles reporting male patients, while 7 articles focused on females. Two articles included both genders, and 1 did not specify the sex of the cases (see Table 2). The overall mean age of the cases was 29.92 years (SD: 24.46). The most common site of scorpion stings was the lower extremities, particularly the feet (Annobil et al., 1991; Aslanyavrusu et al., 2024; Bucaretchi et al., 2016; Majumdar et al., 2020; Naranjo et al., 2021a; Nataraja et al., 2016; Sousa et al., 1995; Uysal et al., 2023) (Table 2).

**Table 2:** Main Characteristics and Clinical Findings of Patients from Analyzed Studies: This table summarizes the primary data from individual case reports and series, including patient age, sex, country of incident, sting location, main symptoms, and imaging findings. The data provides insights into the demographic and clinical profiles of scorpion sting cases associated with ischemic and hemorrhagic strokes.

Age (reference)	Sex	Country	Sting Location	Main symptoms	Imaging findings
3 years (Annobil et al., 1991)	Male	Saudi Arabia	Dorsum of right foot	-Hypothermic -Petechial Hemorrhages -Bilateral crepitations over the lungs -Arterial hypotension -'Flame-shaped’ hemorrhages in both eyes -Papilledema -Pulmonary edema	Cranial CT: bilateral symmetrical peripheral hyperdense areas in both cerebellar hemispheres and parieto-occipital lobes, and white matter areas in the left frontal lobe, in centrum semiovale and anteriorly in the head of left caudate nucleus.
13 years (Groswasser et al., 1991)	Male	Israel	Left hand.	-Diffuse cold sweat -Excessive sahvation -Vomiting -Priapism -Mydriasis -Arterial hypotension -Fever -Lost consciousness -Gangrene -Pulmonary edema	CT scan disclosed mainly biopercular infarct.
1–16 years (mean 8.02) (Nagaraja et al., 1994)	NA	India	N/A	-Hemiplegia -Convulsions -Fever -Dysphasia -Headache -Altered level of consciousness	CT: Infarction was confined to middle cerebral artery territory, often involving basal ganglionic structures and was associated with focal or diffuse atrophy.
9 year (Sousa et al., 1995)	Male	Venezuela	Plantar region of the right foot	-Pulmonary edema -Left hemiplegia -Upper digestive bleeding -Drowsiness -Uncontrollable vomiting -Tachycardia -Tachypnea -Bilateral crackles	CT: hypodense lesion in his right cerebral hemisphere consistent with an ischemic stroke.
8 year (Fernández-Bouzas et al., 2000)	Female	Mexico	N/A	-Vomiting -Sweating -Loss of consciousness -Tachycardia -Hypotension -Fever -Quadriplegia -Alteration of cranial nerves III, VI, VII	MRI: An injury giving low signal on T1- and high signal on T2 weighted images was seen in the pons, larger on the right, extending to the cerebral peduncle.
7 year (Fernández-Bouzas et al., 2000)	Male	Mexico	N/A	-Vomiting -Conversations -Lost consciousness -Stupor -Hemiplegic -Hypotension -Tachycardia -Paralysis of the VII nerve	CT: slight mass effect. MRI: An extensive cortical and subcortical lesion, low-intensity on T1- and high intensity on T2-weighted images, running from the Sylvian region to the occipital lobe of the right hemisphere, producing moderate mass effect.
18–35 years (mean 32) (Udayakumar et al., 2006)	Male	India	N/A	-Hemiparesis -Coma	CT and MRI: frontal intralobar hemorrhage, 1 patient had hemorrhage in the putamen. 2 patients were involved of the middle cerebral artery territory one due to disseminated intravascular coagulation.
35 years (Jain et al., 2006)	Male	India	Left buttock	-Uneasiness -Excessive sweating -Vomiting -Drowsiness -Mydriasis -Hemiparesis	CT (non-enhancing): multiple infarctsinvolving the cerebellar, parieto-occipital and thalamicregions.
18–49 years (mean 35) (Bhattacharya et al., 2008)	Male	India	N/A	-Hemiparesis -Hypertension	CT and MRI: intraventricular hemorrhage at 1 patient, hemorrhage in the putamen 1 patient, involvement of the MCA territory - due to disseminated intravascular coagulation 1 patient.
0.5–90 years (mean 14.7) (Bouaziz et al., 2008)	Female/Male	Tunisia	-Feet -Hand -Chest -Head (The study does not specify exactly the sting sites of the stroke patients)	-Impaired consciousness -Coma -Convulsion -Agitation -Squint -Bilateral myosis -Bilateral mydriasis -Anisocoria -Hyper sweating -Fever -Priapism -Myoclonia (The study does not specify the specific symptoms of stroke patients)	CT: Brain ischemia at 5 patients (50%); brain edema at 2 patients, and brain atrophy at 2 patients.
8 month (Sığırcı et al., 2014)	Female	Turkey	Left side of neck	-Vomiting -Sweating -Seizures -Hypotension -Tachycardia	MRI: focal intensities in cerebellum and extensive cortical and subcortical intensities in cerebrum including corpus callosum on T2-weighted image that caused bright signal on diffusion-weighted imaging with decreased apparent diffusion coefficient.
3 years (Prasad et al., 2014)	Female	India	Back	-Sweating -Seizures -Hypotension -Tachycardia -Loss of consciousness -Aphasic -Weakness	CT: infarction in right and left frontoparietal lobes.
83 years (Eze et al., 2014)	Female	Nigeria	Left foot	-Speech difficulty -Alteration of sensorium -Weakness -Facial nerve palsy -Hemiplegia	CT: acute left intracerebral hemorrhage in the fronto-temporal region with attendant brain shift.
44 years (Bucaretchi et al., 2016)	Female	Brazil	Left foot	-Vomiting -Pallor -Confusion -Hypotension	Brain CT: diffuse bilateral cerebellar hypodensity, with partial involvement of both occipital lobes and thalamus, obstructive hydrocephaly with signs of cerebrospinal fluid extravasation, and ascending transtentorial herniation, suggestive of bilateral ischemia involving the posterior cerebral circulation.
25 years (Nataraja et al., 2016)	Female	India	Right foot	-Breathlessness -Weakness of right upper and lower limbs -Slurring of speech -Left upper motor neuron type facial palsy -Deep tendon reflexes diminished	MRA: diffusion weighted imaging of brain showed an acute infarct in the right capsuloganglionic region.
32 years (Nataraja et al., 2016)	Female	India	N/A	-Breathlessness - Left sided ptosis -Drowsiness - Third nerve palsy -Hemiparesis	CT: acute hemorrhage in left frontal region with mild mass effect.
54 years (Reddy et al., 2017)	Female	India	Tip of digit 3 of left hand	-Dyspnea -Palpitations -Tachycardia -Hypotension -Paucity of movements	MRI: large MCA territory ischemic stroke involving the cortical and subcortical areas.
Mean 40.6 years (Mishra et al., 2018)	Male	India	N/A	-Sweating -Palpitations -Hypertension	CT and MRI: CT cerebrovascular injury.
21 years (Nagar et al., 2018)	Male	India	Flexure aspect of right forearm	-Vomiting -Sweating -Dyspneic -Cough -Altered sensorium -Hypertension -Tachycardia -Tachypnea -Right upper and lower limb weakness -Reflexes decreased on right side	Non-contrast CT: multiple infarcts involving bilateral cerebellar hemisphere, bilateral occipital lobes, medulla and Pons on right side, right temporal lobe, and right thalamus, left parietal lobe.
69 years (Bordón et al., 2018)	Women	Argentina	Left foot	-Hypertension -Tachycardia -Tachypnea -Sweating -Hypothermic	CT: cortical and subcortical left temporo parieto occipital hematoma with extension to the subarachnoid space and in caudal direction to the basal ganglia and internal capsule.
40 years (Majumdar et al., 2020)	Male	India	Right foot	-Headache -Vomiting -Weakness of the right side of the body -Loss of consciousness -Arterial hypertension -Cellulitis of the right foot	Non-contrast CT: revealed intracerebral hemorrhages in left temporoparietal lobe extending into left basal ganglia with intraventricular extension.
2 years (Naranjo et al., 2021a)	Male	Venezuela	Second right toe	-Pancreatitis -Pulmonary edema - Respiratory distress - Cardiomegaly	CT: hypodense lesion in his right cerebral hemisphere consistent with an ischemic stroke.
60 years (Ravi and Kandan, 2023)	Male	India	Right ring finger	- Loss of vision -Altered sensorium -Disorientation -Blackish discoloration of the distal part of the right ring finger	Non-Contrast CT brain: hypodense lesion in the bilateral basal ganglia, and bilateral occipital lobe consistent with ischemic stroke.
8 years (Imad et al., 2023)	Male	Marrocco	N/A	-Cardiogenic shock -Tachycardia -Hypotension -Fever -Consciousness disorders -Aphasia -Right-sided hemiplegia	CT scan with contrast: ischemic stroke in the territory of the left middle cerebral artery.
62 years (Uysal et al., 2023)	Male	Turkey	Right leg	-Chills -Shivering -Sweating -Nausea -Vomiting -Fluctuations in consciousness -Headache -Neck stiffness	Noncontract head CT: hyperdense foci in both posterior parietal lobes suggestive of subarachnoid hemorrhage.
69 years (Aslanyavrusu et al., 2024)	Male	Turkey	Left leg	-Weakness -Speech disorder -Dysarthria -Facial asymmetry -Loss of strength on the left side	No significant pathology was observed in MRI, brain CT, and brain CT angiography (it was thought that there was a transient ischemic attack due to scorpion venom).

CT: computed tomography, MRA: Magnetic resonance angiogram, MCA: middle cerebral artery

### 3.2 Scorpions identified in stroke cases

The most frequently encountered family of scorpions was *Buthidae*, with the genus *Tityus* being the most prevalent, followed by *Leiurus* and *Hottentotta* (Bucaretchi et al., 2016; Groswasser et al., 1991; Majumdar et al., 2020; Naranjo et al., 2021a; Reddy et al., 2017; Sığırcı et al., 2014; Sousa et al., 1995). Another identified family was Diplocentridae, specifically the genus *Nebo* (Annobil et al., 1991). However, the majority of studies (n = 14) did not specify the scorpion type responsible for the stings (Table 1).

Among the Buthidae family, Tityus is known for its potent venom, which can cause severe symptoms and even death in humans. *Leiurus,* often referred to as the “deathstalker,” is similarly notorious for its highly toxic sting, which can lead to intense pain, respiratory issues, and cardiovascular complications (Ghoneim et al., 2020). *Hottentotta*, though less well-known, also poses significant health risks with its venom, potentially causing neurotoxic and cardiotoxic effects.

The *Diplocentridae* family, represented by the genus *Nebo*, is less commonly reported in medical literature but still poses serious health risks (Hendrixson, 2006). The venom of *Nebo* species can cause severe local pain, systemic symptoms, and, in rare cases, more severe complications such as stroke.

### 3.3 Clinical manifestations

In addition to local symptoms such as pain, erythema, and edema, scorpion stings resulted in various clinical manifestations among patients. Neurological symptoms were the most common, including alterations in consciousness, seizures, hemiplegia, hemiparesis, limb weakness (upper or lower), miosis, mydriasis, aphasia, and cranial nerve impairments (III, VI, VII). Other symptoms included confusion, disorientation, neck stiffness, coma, aphasia, decreased reflexes, and facial asymmetry (Aslanyavrusu et al., 2024; Bhattacharya et al., 2008; Bouaziz et al., 2008; Bucaretchi et al., 2016; Fernández-Bouzas et al., 2000; Groswasser et al., 1991; Imad et al., 2023; Jain et al., 2006; Majumdar et al., 2020; Nagar et al., 2018; Nagaraja et al., 1994; Nataraja et al., 2016; Prasad et al., 2014; Ravi and Kandan, 2023; Reddy et al., 2017; Sığırcı et al., 2014; Sousa et al., 1995; Udayakumar et al., 2006; Uysal et al., 2023).

Cardiovascular complications reported included hypotension, hypertension, tachycardia, palpitations, and even cardiogenic shock (Annobil et al., 1991; Bhattacharya et al., 2008; Bucaretchi et al., 2016; Fernández-Bouzas et al., 2000; Groswasser et al., 1991; Imad et al., 2023; Majumdar et al., 2020; Mishra et al., 2018; Nagaraja et al., 1994; Prasad et al., 2014; Reddy et al., 2017; Sığırcı et al., 2014; Sousa et al., 1995). Gastrointestinal symptoms included vomiting, nausea, and upper digestive system bleeding (Bucaretchi et al., 2016; Fernández-Bouzas et al., 2000; Groswasser et al., 1991; Jain et al., 2006; Majumdar et al., 2020; Nagar et al., 2018; Sığırcı et al., 2014; Sousa et al., 1995; Uysal et al., 2023), while respiratory manifestations ranged from tachypnea and pulmonary crepitations to pulmonary edema, cough, respiratory distress, and dyspnea (1–4,13,16,18). Other reported symptoms encompassed gangrene, priapism, vision loss, fever, hypothermia, sweating, petechiae, papilledema, flame-shaped hemorrhages, salivation, pallor, chills, cellulitis, and pancreatitis (Annobil et al., 1991; Fernández-Bouzas et al., 2000; Groswasser et al., 1991; Jain et al., 2006; Majumdar et al., 2020; Mishra et al., 2018; Nagaraja et al., 1994; Naranjo et al., 2021a; Nataraja et al., 2016; Prasad et al., 2014; Ravi and Kandan, 2023; Sığırcı et al., 2014) (Table 2).

### 3.4 Imaging findings

The primary imaging modalities that are used to confirm a stroke after scorpion envenomation are computed tomography (CT) and magnetic resonance imaging (MRI). In the pediatric population, CT findings revealed hyperdense areas in the parieto-occipital lobes and both cerebellar lobes, along with areas of white matter in the frontal lobe, centrum semiovale, and caudate nucleus. Hypodense regions were also observed in the frontoparietal and front-parietal-temporal lobes (Annobil et al., 1991; Imad et al., 2023; Naranjo et al., 2021a; Prasad et al., 2014; Sousa et al., 1995). An identical case of bilateral frontal opercular infarction was also documented (Groswasser et al., 1991).

Regarding MRI findings, extensive cortical and subcortical lesions were observed from the Sylvian region to the right occipital lobe, characterized by low intensity on T1 and high intensity on T2. Additionally, lesions in the pons extended into the cerebral peduncle, showing low signal on T1 and high signal on T2, with focal intensities in the cerebellum and cortical and subcortical areas extending to the corpus callosum (Sığırcı et al., 2014).

In the adult population, CT scans revealed hypodensities in the occipital lobes and thalamus, as well as infarctions involving the cerebellum, parieto-occipital and parietal regions, corona radiata, lentiform nucleus, basal ganglia, and thalamic regions. Hyperdense areas were noted in the parietal lobes, and hemorrhages were observed in the left frontal region and left basal ganglia, extending into the left temporoparietal lobe with intraventricular extension. Asymmetry was noted in the bilateral occipital lobes, right medulla and pons, right temporal lobe, right thalamus, left parietal lobe, and cerebellar hemispheres (Bucaretchi et al., 2016; Jain et al., 2006; Majumdar et al., 2020; Nagar et al., 2018; Nataraja et al., 2016; Ravi and Kandan, 2023; Uysal et al., 2023). MRI findings in adults included ischemic strokes involving the middle cerebral artery territory, affecting cortical and subcortical areas, and subarachnoid hemorrhages (Bhattacharya et al., 2008; Reddy et al., 2017; Uysal et al., 2023).

## 4 Discussion

This systematic review shows that the most common manifestations after a scorpion sting include local discomfort such as pain, edema, and erythema. However, systemic complications such as allergic reactions, pancreatitis, renal failure, acute respiratory failure, and systemic inflammatory response syndrome can also occur. Approximately 2% of complications involve the central nervous system, and an additional 8% are related to cerebrovascular problems (Eze et al., 2014; Nataraja et al., 2016; Uysal et al., 2023). Damage to the nervous system may occur through several mechanisms, including systemic arterial hypertension, reduced carotid arterial blood flow (Karnad, 1998; Naranjo et al., 2021a), direct effects of toxins causing encephalopathies, or direct damage to the endothelium causing vasculitis (Gowtham et al., 2022).

Scorpion venom primarily consists of neurotoxins that depolarize nerve cell membranes through various mechanisms: β-toxins open sodium channels, α-toxins inhibit sodium channel deactivation, and kappa-neurotoxins block potassium channels. Additionally, calcines act as agonists of ryanodine receptors, increasing intracellular Ca^2+^ levels and causing contractile paralysis (Ahmadi et al., 2020; Del Brutto, 2013). Other effects include the stimulation of alpha-adrenergic receptors, leading to hypertension, tachycardia, myocardial dysfunction, and pulmonary edema (Udayakumar et al., 2006).

**Fig.3:**
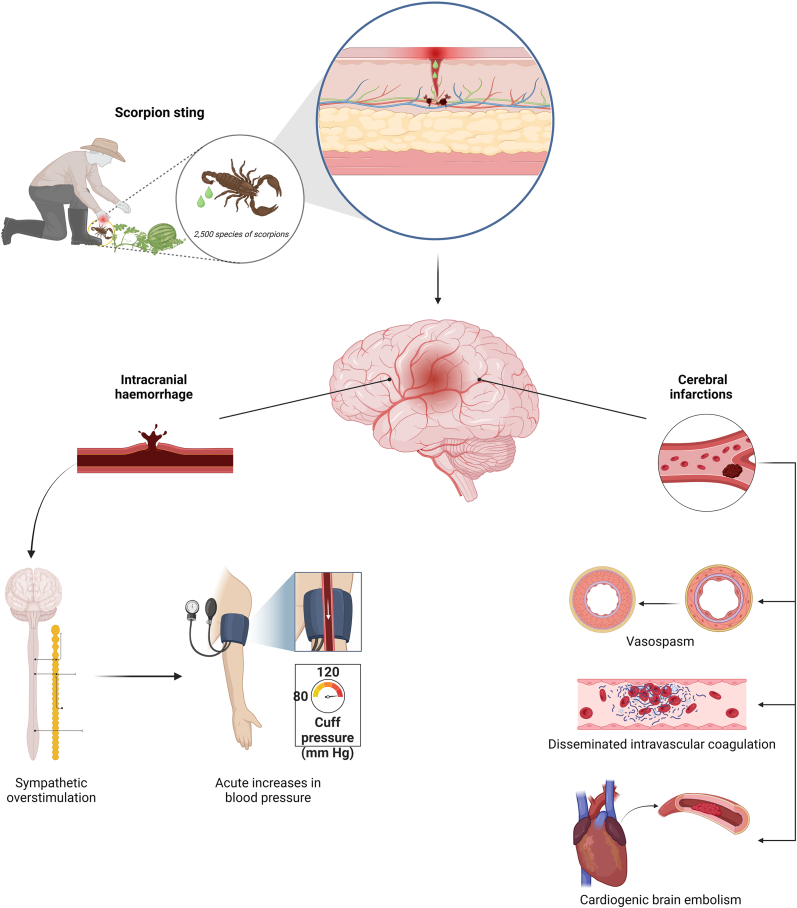
Main mechanisms by which stroke occurs due to the scorpion sting.

Several mechanisms have been identified through which a scorpion sting can cause stroke (Fig. 3). In hemorrhagic cases, sympathetic overstimulation can lead to a sudden increase in blood pressure, potentially rupturing perforating arteries (Del Brutto and Del Brutto, 2013). Cerebral hypoperfusion-related cases highlight disseminated intravascular coagulation, which increases platelet aggregation, excess catecholamines inducing endothelin increase and subsequent vasospasm, and cardiogenic cerebral embolism resulting from myocarditis (Bhattacharya et al., 2008; Del Brutto, 2013; Reddy et al., 2017). The venom's vasculotoxicity damages endothelial cells and causes vasculitis. Moreover, increased acetylcholine levels due to scorpion venom effects lead to excessive sweating and vomiting, contributing to hypotension. This, combined with carotid vasospasm, can exacerbate cerebral ischemia (Eze et al., 2014; Prasad et al., 2014). Finally, depressed left ventricular function is another potential mechanism for stroke development (Fernández-Bouzas et al., 2000).

Living in rural areas is the main risk factor for scorpion stings. In countries like India, scorpion stings pose a significant public health challenge in rural settings (Nataraja et al., 2016). Rural environments often require storing firewood, leaves, tools, or construction materials near homes, creating ideal habitats for scorpions. A study in Mexico showed that handling firewood increases sting risk due to prolonged field exposure and ground interaction (Trinidad-Porfirio et al., 2023). Poultry farming in rural areas also creates ideal scorpion habitats, with straw from nests providing shelter and increasing human contact risks (Trinidad-Porfirio et al., 2023). The lack of secure housing and proximity to scorpion habitats in rural areas facilitate their entry into homes and increase human interaction opportunities (Ebrahimi et al., 2017).

Symptomatic management, life support, and venom neutralization are key to managing scorpion stings. Antivenom administration within the first few hours’ post-sting is recommended. Clinical conditions often rapidly improve with timely antivenom administration, with plasma venom levels typically becoming undetectable within an hour of treatment initiation (Boyer Leslie V. et al., 2009; Ochoa-Andrade et al., 2022). The use of prazosin, an alpha-1 adrenergic receptor antagonist, has been shown to reduce mortality, and early administration can prevent cerebrovascular complications from scorpion envenomation (Biswal et al., 2006; Ochoa-Andrade et al., 2022; Prasad et al., 2014).

From a public and global health perspective, rural and marginalized remote areas are disproportionately affected by scorpion stings. It is our responsibility to generate calls to action to train local doctors and healthcare providers to manage these stings effectively. Enhanced training and resources in these areas can significantly reduce the burden of scorpion stings, improve patient outcomes, and prevent severe complications like stroke. Efforts must be made to ensure that medical professionals in these regions are equipped with the necessary knowledge and tools to respond promptly and effectively to scorpion envenomation cases, ultimately reducing morbidity and mortality rates associated with these incidents.

## 5 Limitations

One of the main limitations of this study is the reliance on case reports, which do not establish causal relationships. Additionally, the nature of these studies does not allow for identifying risk factors predisposing individuals to stroke following a scorpion sting. Focusing exclusively on stroke may have caused us to overlook other neurological complications related to scorpion stings. A significant limitation was the frequent lack of identification of the responsible scorpion species in the articles reviewed, hindering conclusions about which species are more likely to cause strokes. Furthermore, restricting the literature search to English and Spanish may have resulted in the omission of relevant studies published in other languages.

## 6 Conclusion

This systematic review underscores the importance of recognizing the potential for severe neurological complications, including strokes, following scorpion stings. Early diagnosis and intervention are crucial in improving patient outcomes. Healthcare providers, especially in regions where scorpions are prevalent, should be trained to recognize and manage these symptoms promptly. Enhanced awareness and training can lead to better preparedness and response, ultimately reducing the morbidity and mortality associated with scorpion stings.

Furthermore, efforts should be made to identify the specific scorpion species responsible for stings to understand better the risks associated with different species and to develop targeted prevention and treatment strategies. Expanding research to include studies in multiple languages and broadening the scope to encompass all neurological complications can provide a more comprehensive understanding of the health impacts of scorpion envenomation.

## Ethics approval and consent to participate

This study did not require ethics approval as it involved the synthesis of previously published data and complied with international harmonization and ethical guidelines.

## Consent for publication

Not applicable.

## Availability of data and materials

Not applicable, as primary data was not generated for this study.

## Funding

This research was supported by 10.13039/100021068Universidad de Las Américas, project MED.EOP.23.01.

## Ethical statement

The work was carried out based on the review of articles obtained through public access databases, none of the data used can be identified with personal information since the information in the papers did not include names, addresses, emails, locations or telephone numbers, therefore, to carry out this work, the approval of the Institutional Review Board or the informed consent of patients was not required.

## CRediT authorship contribution statement

**Jorge Vasconez-Gonzalez:** Writing – original draft, Visualization, Validation, Resources, Methodology, Investigation, Formal analysis, Data curation, Conceptualization. **Karen Delgado-Moreira:** Writing – original draft, Validation, Resources, Methodology, Investigation, Data curation, Conceptualization. **Esteban Gamez-Rivera:** Writing – original draft, Visualization, Validation, Resources, Methodology, Investigation, Data curation. **María Belen Lopez-Molina:** Writing – original draft, Validation, Resources, Methodology, Investigation, Formal analysis, Data curation. **Fredy Lizarazo Davila:** Writing – original draft, Visualization, Resources, Methodology, Investigation, Data curation. **Juan S. Izquierdo-Condoy:** Writing – review & editing, Visualization, Validation, Supervision, Resources, Methodology, Investigation, Formal analysis, Data curation. **Esteban Ortiz-Prado:** Writing – review & editing, Validation, Supervision, Resources, Project administration, Methodology, Investigation, Formal analysis.

## Declaration of competing interest

The authors declare that they have no known competing financial interests or personal relationships that could have appeared to influence the work reported in this paper.

## Figures and Tables

**Table S1:** NEWCASTLE - OTTAWA QUALITY ASSESSMENT SCALE

Study	1	2	3	4	5	6	7	8	Score
Nagaraja et al. (Nagaraja et al., 1994)	*		*	*	**	*	*	*	8
Bouaziz et al.(Bouaziz et al., 2008)	*		*	*	*	*	*	*	7

**Note:** A study can receive a maximum of one star for each item numbered within the Selection and Result categories. A maximum of two stars can be awarded for comparability.

**Table S2:** JBI Critical Appraisal Checklist for Case Reports

Study	1	2	3	4	5	6	7	8	Score
Annobil et al. (Annobil et al., 1991)	YES	YES	YES	YES	YES	YES	YES	YES	8
Groswasser et al.(Groswasser et al., 1991)	NO	YES	YES	YES	YES	YES	YES	YES	7
Sousa et al.(Sousa et al., 1995)	YES	YES	YES	YES	YES	YES	YES	YES	8
Fernández-Bouzas et al.(Fernández-Bouzas et al., 2000)	YES	YES	YES	YES	YES	YES	YES	YES	8
Jain et al.(Jain et al., 2006)	NO	YES	YES	YES	YES	YES	YES	YES	7
Sığırcı et al.(Sığırcı et al., 2014)	YES	YES	YES	YES	YES	YES	YES	YES	8
Prasad et al. (Prasad et al., 2014)	YES	YES	YES	YES	YES	YES	YES	YES	8
Eze et al. (Eze et al., 2014)	YES	YES	YES	YES	YES	YES	YES	YES	8
Bucaretchi et al.(Bucaretchi et al., 2016)	YES	YES	YES	YES	YES	YES	YES	YES	8
Nataraja et al. (Nataraja et al., 2016)	YES	YES	YES	YES	YES	YES	YES	YES	8
Reddy et al. (Reddy et al., 2017)	YES	YES	YES	YES	YES	YES	YES	YES	8
Nagar et al.(Nagar et al., 2018)	YES	YES	YES	YES	YES	YES	YES	YES	8
Bordon et al. (Bordón et al., 2018)	YES	YES	YES	YES	YES	YES	YES	YES	8
Majumdar et al.(Majumdar et al., 2020)	YES	YES	YES	YES	YES	YES	YES	YES	8
Naranjo et al.(Naranjo et al., 2021a)	NO	YES	YES	YES	YES	YES	YES	YES	7
Ravi & Kanda (Ravi and Kandan, 2023)	YES	YES	YES	YES	YES	YES	YES	YES	8
Imad et al.(Imad et al., 2023)	YES	YES	YES	YES	YES	YES	YES	YES	8
Uysal et al. (Uysal et al., 2023)	YES	YES	YES	YES	YES	YES	YES	YES	8
Aslanyavrusu et al. (Aslanyavrusu et al., 2024)	YES	YES	YES	YES	YES	YES	YES	YES	8

1. Were patient demographics clearly described?

2. Was the patient's history clearly described and presented as a timeline?

3. Was the patient's current clinical condition clearly described at the time of presentation?

4. Were diagnostic tests or evaluation methods and results clearly described?

5. Were interventions or treatment procedures clearly described?

6. Was the post-intervention clinical condition clearly described?

7. Were adverse (harm) or unforeseen events identified and described?

8. Does the case report provide lessons to take away?

Quality scores were categorized into three groups: Low: 1–4, Moderate: 5–7, and High: >8

**Table S3:** JBI Critical Appraisal Checklist for Case Series

Study	1	2	3	4	5	6	7	8	9	10	Score
Udayakumar et al.(Udayakumar et al., 2006)	YES	YES	YES	YES	YES	YES	YES	YES	YES	YES	10
Bhattacharya et la. (Bhattacharya et al., 2008)	YES	YES	YES	YES	YES	YES	YES	YES	YES	YES	10
Mishra et al.(Mishra et al., 2018)	YES	YES	YES	YES	YES	NO	NO	NO	NO	NO	5

1. Were there clear criteria for inclusion in the case series?

2. Was the condition measured in a standard and reliable way for all participants included in the case series?

3. Were valid methods of condition identification used for all participants included in the case series?

4. Did the case series include consecutive participants?

5. Did the case series have complete inclusion of participants?

6. Were there clear reports on the demographics of the study participants?

7. Was there a clear reporting of the clinical information of the participants?

8. Were outcomes or case follow-up results clearly reported?

9. Was there clear reporting of demographic information from the presenting sites/clinics?

10. Was the statistical analysis appropriate?

Quality scores were categorized into three groups: Low: 1–4, Moderate: 5–7, and High: >8

## Data Availability

No data was used for the research described in the article.

## Supplementary material

### Selection

1. Representativeness of the exposed court.
2. Selection of the unexposed court.
3. Exposure determination.
4. Demonstration that the current outcome of interest was not present at baseline.

### Comparability

5. Cohort comparability based on design or analysis.

### Results

6. Evaluation of the result.
7. Was the follow-up long enough for the results to occur?
8. Adequacy of cohort follow-up.

### Interpretation

Good quality: 3 or 4 stars in the selection domain and 1 or 2 stars in the comparability domain and 2 or 3 stars in the outcome/exposure domain.

Acceptable quality: 2 stars in the selection domain and 1 or 2 stars in the comparability domain and 2 or 3 stars in the outcome/exposure domain.

Poor quality: 0 or 1 star in the selection domain or 0 stars in the comparability domain or 0 or 1 stars in the outcome/exposure domain.
